# Artificial Intelligence-Based Application to Explore Inhibitors of Neurodegenerative Diseases

**DOI:** 10.3389/fnbot.2020.617327

**Published:** 2020-12-22

**Authors:** Leping Deng, Weihe Zhong, Lu Zhao, Xuedong He, Zongkai Lian, Shancheng Jiang, Calvin Yu-Chian Chen

**Affiliations:** ^1^Artificial Intelligence Medical Center, School of Intelligent Systems Engineering, Sun Yat-sen University, Shenzhen, China; ^2^Department of Clinical Laboratory, The Sixth Affiliated Hospital, Sun Yat-sen University, Guangzhou, China; ^3^Department of Medical Research, China Medical University Hospital, Taiwan, China; ^4^Department of Bioinformatics and Medical Engineering, Asia University, Taiwan, China

**Keywords:** artificial intelligence, deep belief network, molecular dynamic simulation, galectin-3, neurodegenerative disease

## Abstract

Neuroinflammation is a common factor in neurodegenerative diseases, and it has been demonstrated that galectin-3 activates microglia and astrocytes, leading to inflammation. This means that inhibition of galectin-3 may become a new strategy for the treatment of neurodegenerative diseases. Based on this motivation, the objective of this study is to explore an integrated new approach for finding lead compounds that inhibit galectin-3, by combining universal artificial intelligence algorithms with traditional drug screening methods. Based on molecular docking method, potential compounds with high binding affinity were screened out from Chinese medicine database. Manifold artificial intelligence algorithms were performed to validate the docking results and further screen compounds. Among all involved predictive methods, the deep learning-based algorithm made 500 modeling attempts, and the square correlation coefficient of the best trained model on the test sets was 0.9. The XGBoost model reached a square correlation coefficient of 0.97 and a mean square error of only 0.01. We switched to the ZINC database and performed the same experiment, the results showed that the compounds in the former database showed stronger affinity. Finally, we further verified through molecular dynamics simulation that the complex composed of the candidate ligand and the target protein showed stable binding within 100 ns of simulation time. In summary, combined with the application based on artificial intelligence algorithms, we unearthed the active ingredients 1,2-Dimethylbenzene and Typhic acid contained in *Crataegus pinnatifida* and *Typha angustata* might be the effective inhibitors of neurodegenerative diseases. The high prediction accuracy of the models shows that it has practical application value on small sample data sets such as drug screening.

**Graphical Abstract:**
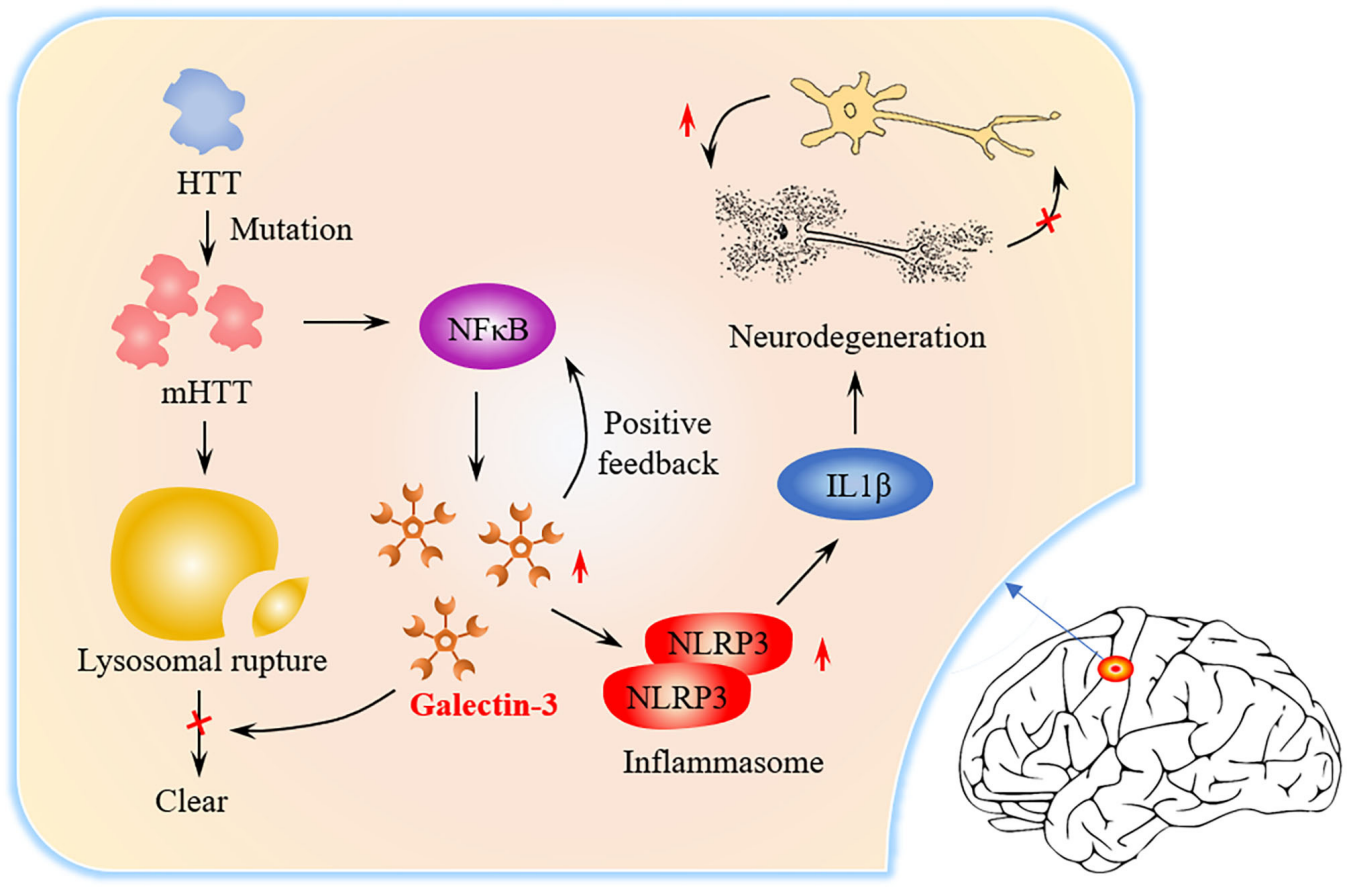
Graphical abstract of the role of Gal3 in HD pathogenesis. The level of Gal3 expressed by microglia is low under normal conditions. In HD patients, mutant Huntingtin (mHTT) continues to accumulate due to Huntingtin (HTT) mutations and NFκB is activated. NFκB triggers Gal3 aggregation, while Gal3 feedback promotes NFκB activation. mHTT causes lysosome damage, but Gal3 prevents the damaged lysosome from being cleared. NLRP3Inflammasome and proinflammatory factors (such as IL1β) increase in number, causing neuronal death and repair of damage.

## Introduction

Neurodegenerative diseases (ND) cause the progressive death of central neurons, leading to brain dysfunction and the development of diseases, such as Huntington's disease (HD) (Macdonald et al., 1993), Alzheimer's disease (AD) (McKhann et al., 1984) and Parkinson's disease. ND often result from the aberrant deposition of aggregated host proteins (Voet et al., 2019). At present, the mechanism of ND is not clear, but inflammation is considered to be a common factor (Saijo et al., 2010). Galectin-3 (Gal3) is an important member of the galectin family (Romero and Gabius, 2019). Gal3 is a key molecule linking inflammation and decreased insulin sensitivity (Li et al., 2016). Recently, more and more studies have shown that Gal3 is closely related to ND. Gal3 plays an important role in regulating inflammation (Henderson and Sethi, 2009). Extensive research on Gal3 in the central nervous system has shown that Gal3 promotes inflammation (Shin, 2013). Inhibition of Gal3 can help reduce inflammation in ND (Ramirez Hernandez et al., 2020). In the brains of AD patients, Gal3 promotes the activation of microglia (Ramirez et al., 2019), and inhibition of Gal3 may be a potential pharmacological method for the treatment of AD (Boza-Serrano et al., 2019). The latest research found that the brain Gal-3 content of patients and mice with HD is higher than normal. Inflammation can be controlled and the accumulation of mutant Huntingtin is reduced by inhibiting Gal3 (Siew et al., 2019). From the signal transmission process (Graphical Abstract), it can be identified that inhibition of Gal3 may become a new drug target for HD treatment.

Understanding the basic laws of target protein-drug interactions is the basis of molecular targeted drug design, which plays a vital role in drug discovery and drug design (Rahman et al., 2020). Gal3 is one of the most potential target proteins for treating ND. Molecular docking method and active ingredient screening techniques are used to screen out drug molecules that have inhibitory effects on the target protein from drug database (Abdolmaleki et al., 2017). Traditional Chinese medicine is a medicine with great modern potential (Wen et al., 2019). Greater than 85 percent of patients diagnosed with COVID-19 in China have received Chinese medicine for adjuvant treatment (Yang et al., 2020). Therefore, discovering and designing the Chinese medicine prescription inhibitors of Gal3 is expected to have curative effects ND treatment. With the continuous improvement of computer performance, artificial intelligence (AI)-based methods are increasingly applied to various stages of drug discovery (Chen et al., 2018a; Schneider et al., 2020; Senior et al., 2020). Machine learning methods are used to predict biologically active properties (Kaiser et al., 2018; Correia et al., 2020). SVM and other methods were used to establish four quantitative prediction models of the inhibitory activity value of HIV-1 integrase inhibitors (Xuan et al., 2013). The DeepTox algorithm shows good accuracy in predicting the toxicity of compounds (Mayr et al., 2016). Drug-drug interactions prediction (Zhang et al., 2019), biomolecular properties prediction (Hessler and Baringhaus, 2018) and quantum mechanical property prediction are combined with AI that is used in pharmacodynamic research of new synthetic drug candidates, which can greatly save costs. The combination of AI and traditional Chinese medicine may be a new development trend of modern Chinese medicine in the future (Liu et al., 2017).

**Figure 1:**
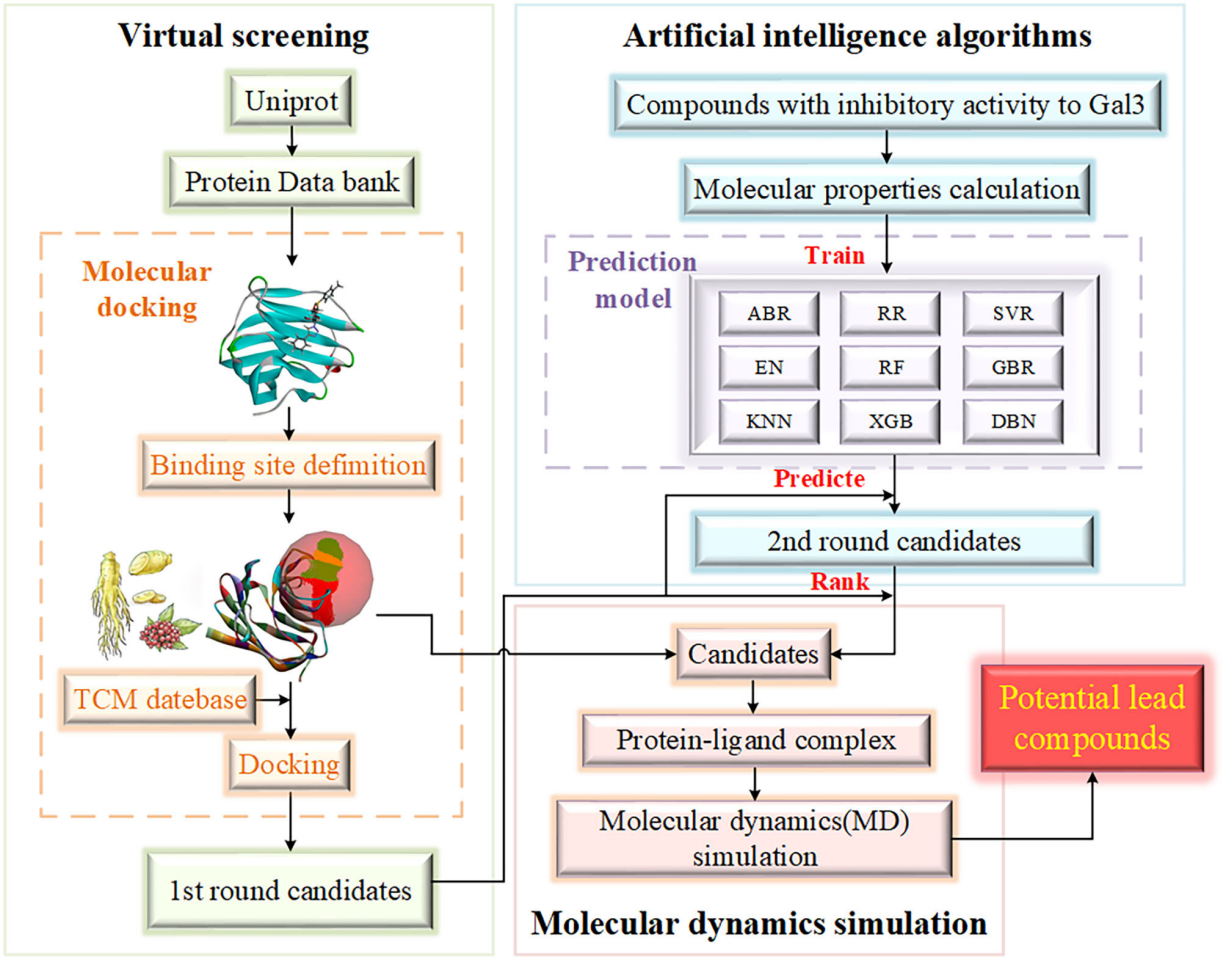
The flowchart of experiment design. The experiment is mainly divided into three parts, including virtual screening, artificial intelligence algorithms and molecular dynamics simulation. The range of candidates is gradually compressed.

Based on the discovery of Gal3 as a key target protein in a new pathogenesis closely related to HD, the purpose of this study to screen potential compounds that inhibit Gal3 has been determined. The contribution of our study is 2-fold. Methodologically, we have added AI algorithms to establish a compound activity value prediction model based on traditional drug screening methods, including molecular docking and molecular dynamics (MD) simulation (Kumar et al., 2020), accelerating the process of new drug discovery. Screening drug molecules from traditional Chinese medicine (TCM) database is an innovation and beneficial supplement to screening from the general database. Practically, the underlying relationship between compound activity values and input molecular properties can be acquired through algorithm models. Target variable is an important evaluation index of drugs, which can provide researchers with reference, and the high accuracy of the model improves reliability. Figure 1 provides the flowchart of experiment design.

## Materials and Methods

### Molecular Docking Screening

Molecular docking is a virtual screening tool and can identify lead compounds from large small molecule databases, which reducing the number of experimental screening compounds and thus shortening the research cycle (Elmezayen et al., 2020; Pant et al., 2020). Molecular docking can be used to investigate the possible weak interactions between small molecule ligands and large molecule receptors and to calculate their affinity (Liu et al., 2019). The sequence of Gal3 was obtained from UniProt knowledgebase [(Identifier: P17931) (Bateman et al., 2017), and the crystal structure was obtained from RCSB Protein Data Bank (PDB ID: 6QLR) (Burley et al., 2019; Kumar et al., 2019), with a resolution of 0.97 Å. Through preprocessing operation, including removing crystal water molecules in the composite crystals, replenishing missing hydrogen atoms and optimizing energy by using the CHARMm27 force field (Brooks et al., 2009), a receptor protein with high confidence binding site was presented. The binding site was defined with the pro-ligand. A total of 18,776 molecule compounds obtained from the TCM database (TCM Database @Taiwan) (Chen, 2011) and 148,120 molecule compounds from ZINC database (http://zinc.docking.org/) (Irwin and Shoichet, 2005) were used as ligands for molecular docking, respectively. The original ligand in the complex was used as the control ligand, which was used as a reference for docking results. All involved experiments were implemented on LigandFit module in Discovery Studio Client v17.2.0.16349 (DS). Ligandfit has the functions of automatic search and confirmation of the active site of the receptor molecule, conformationally flexible multi-ligand docking, and evaluation of interaction scores based on force fields (Venkatachalam et al., 2003).

### Artificial Intelligence-Based Prediction Models

#### Data Collection and Processing

Relevant information (structural formula and IC50) of the small molecules reported that have inhibitory effect on Gal3 were collected from literatures, invention patents, and drug generation companies. Chemdraw was used to draw the structural formula of molecules(Mills, 2006). In addition, from open source small molecule databases such as PubChem (Kim et al., 2019), ChEMBL (Gaulton et al., 2017) and ZINK, we have downloaded the corresponding three-dimensional structure containing Gal3 inhibitors. All collected compounds were saved as Mol format files and converted to SDF format through Chem3D. The sample with clear IC50 value could be kept, and 56 molecular samples were included. Chem3D software was used to minimize the molecular posture energy of all molecule samples. All qualified molecular samples were also subject to molecular attitude energy minimization processing. The IC50 value was changed to pIC50 as target variable by equation (1). All collected and sorted sample molecules were imported into DS to calculate molecular properties of 204 types that used as the input feature set.

(1) $$\begin{array}{r} {pIC}_{50}=6-log_{10}\;\left({IC}_{50}\right) \end{array}$$

We performed AdaBoost, random forest, XGBoost, deep belief network and other models to build models for predicting pIC50 values. These models are widely used AI-based machine learning models, with fast convergence speed and advantages in processing small sample data, which is very suitable for our small-scale data experiments.

#### AdaBoost Model

**Figure 2:**
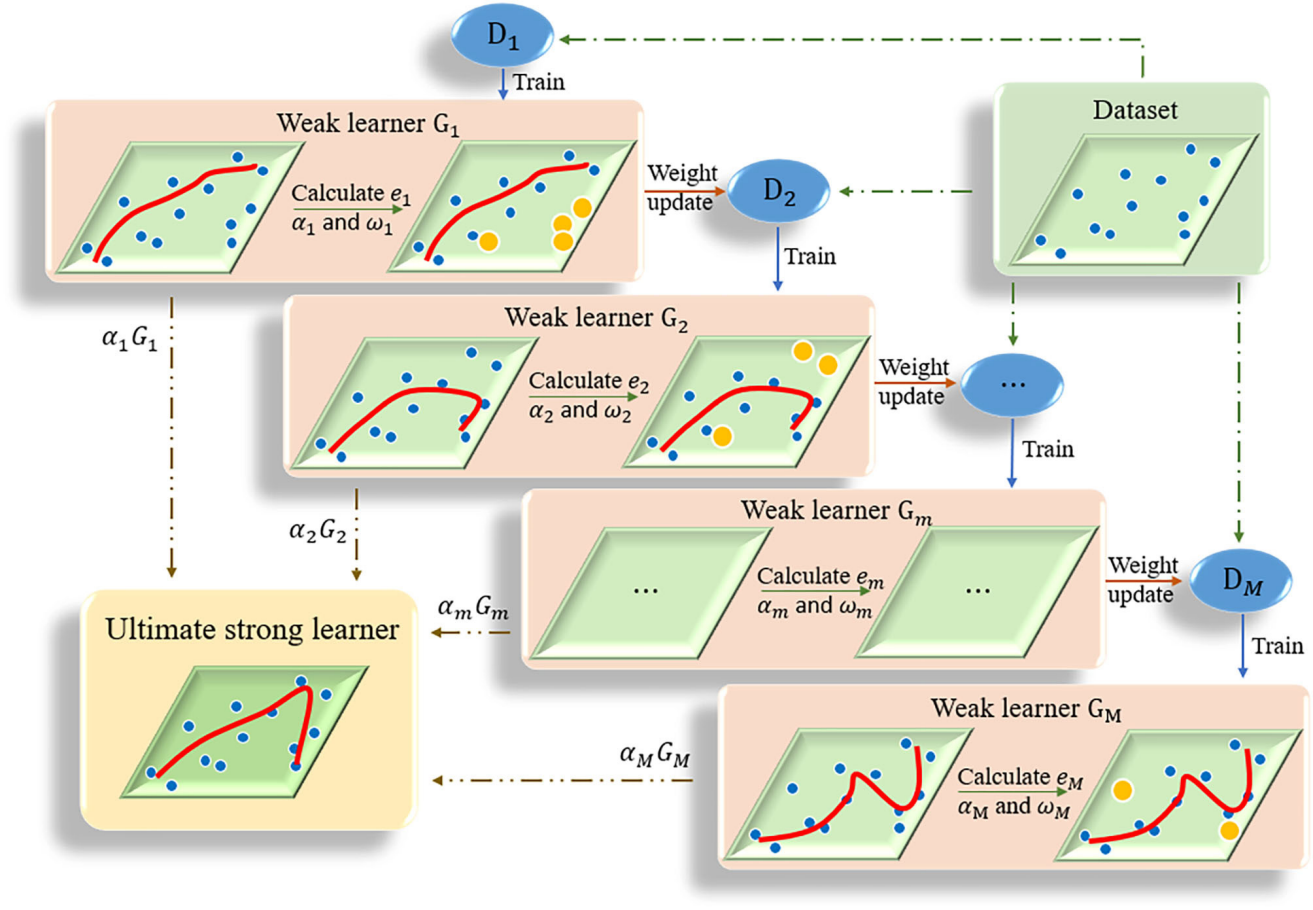
The flow chart of AdaBoost algorithm. Multiple weak learners are combined into one strong learner.

The kernel of adaptive boosting algorithm (AdaBoost) is to build a strong learner by connecting multiple weak learners (Huang et al., 2020). AdaBoost algorithm adjusts the weight of samples in the training set of each round by increasing the weight of the samples that were incorrectly predicted in the previous round (Ratsch et al., 2001). The iteration continues until the predetermined error rate is reached or the specified maximum number of iterations is reached (Figure 2). The ultimate strong learner is combined by linearly weighting and summing all base learners, and the base learner with small error rate has a larger weight coefficient. Details of the AdaBoost algorithm is presented in Algorithm 1. In this study the optimal number of decision trees was set as 15 based on some initial trials.

**Algorithm 1:** AdaBoost

1. Initialize weights distribution of training samples: *D*_1_ = (ω_11_, ω_12_, ...ω_1*i*_..., ω_1*N*_), ω_1*i*_ = 1/*N, i* = 1, 2...., *N*
2. For *m* = 1, 2, ⋯ , *M* multiple iterations: (1) Training the weighted *D*_*m*_ sample set to obtain the base learner *G*_*m*_(*x*) (2) Calculating the maximum error of the training set: *E*_*m*_ = *max*∣*y*_*i*_ − *G*_*m*_(*x*_*i*_)∣ (3) Calculating the relative error of each sample: $e_{mi}=\frac{|y_{i}-G_{m}\left(x_{i}\right)|}{E_{m}}$ (4) Calculating the regression error rate: $e_{m}=\overset{N}{\underset{i=1}{\sum }}\omega_{mi}e_{mi}$ (5) Calculating the weight coefficients of weak learners: $\alpha_{m}=\frac{e_{m}}{1-e_{m}}$ (6) Updating the weight distribution of the sample set: $\omega_{m+1,i}=\frac{\omega_{mi}}{Z_{m}}{\alpha_{m}}^{1-e_{m}}$ $Z_{m}=\overset{N}{\underset{i=1}{\sum }}\omega_{mi}\alpha_{m}^{1-e_{mi}}$ *D*_*m*+1, *i*_ = (ω_*m*+1, 1_, ω_*m*+1, 2_, ...ω_*m*+1, *i*_..., ω_*m*+1, *N*_)
3. Output the ultimate strong learner $f\left(x\right)=\sum_{m=1}^{M}(\ln\frac{1}{\alpha_{m}})\alpha_{m}G_{m}\left(x\right)$ end

#### Ridge Regression Model

Ridge regression (RR) works well on condition that the number of independent variables is more than the sample size. As shown in equation 2, the RR model adds a penalty term of the L2 norm to the objective function of the ordinary linear regression mode (Yang and Wen, 2018), which contributed to the biased estimation of the regression coefficient β. Generally, RR is a regression method that solves the ill-conditioned matrix problem at the cost of giving up unbiasedness and reducing accuracy. The alpha parameter was set to 0.05 in the case study.

(2) $$\begin{array}{r} J\left(\beta \right)={\left(y-X\beta \right)}^{T}+\lambda \beta^{T}\beta \\ \Rightarrow \beta ={\left(X^{T}X+\lambda I\right)}^{-1}X^{T}y \end{array}$$

#### SVM Model

**Figure 3:**
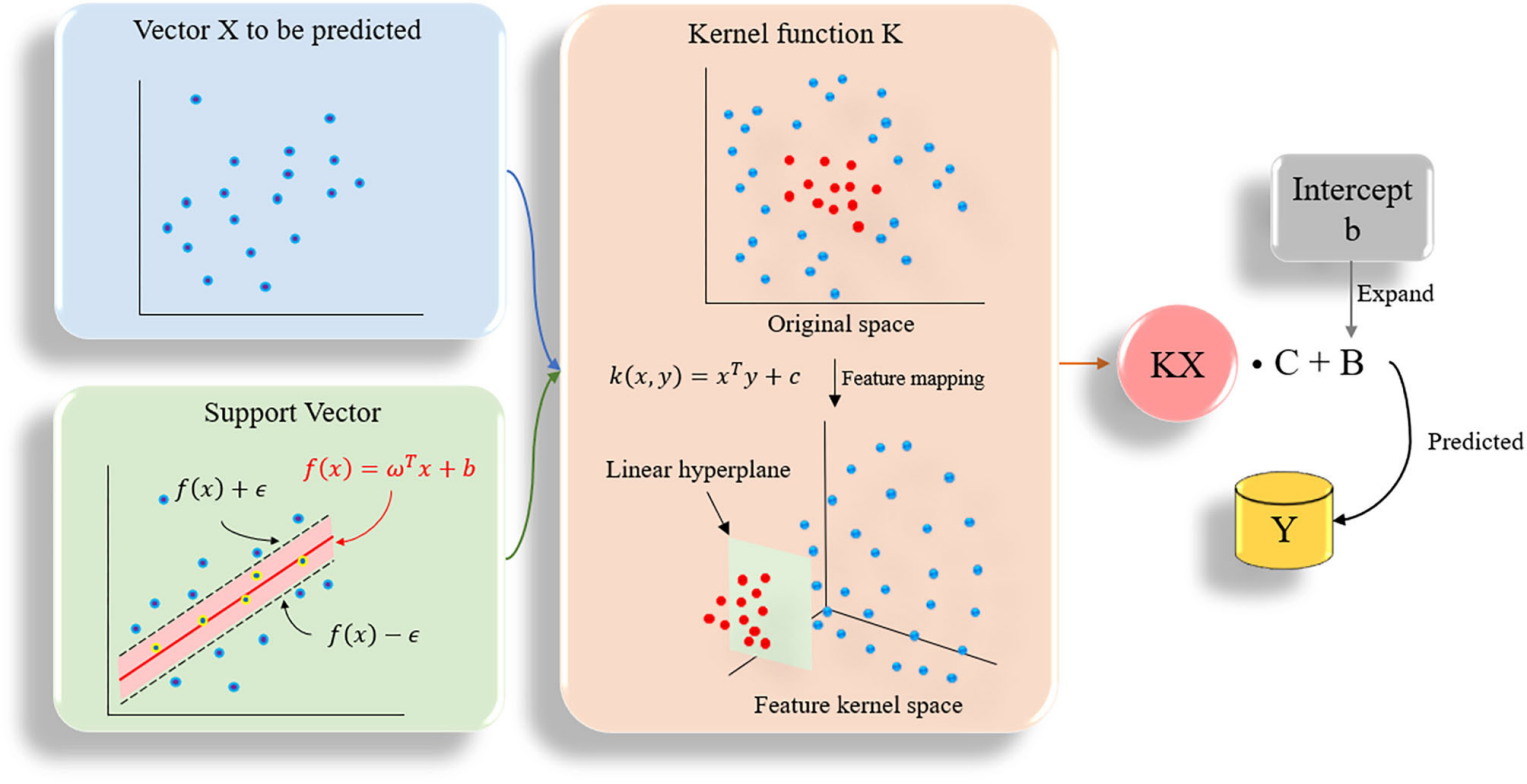
The flow chart of SVR algorithm. All data are plotted in n-dimensional (*n* = 28) space, *n* represents the number of features of the data.

Support vector machine (SVM) can be divided into support vector classification (SVC) and support vector regression (SVR) in practical applications (Chang and Lin, 2011). SVR is designed to fit each training sample and retain all the main features that characterize the algorithm to minimize errors. The kernel function is used to replace the linear term in the linear equation to make the original linear algorithm non-linear, which is used to achieve non-linear regression (Figure 3). SVR has high accuracy and strong generalization ability to solve small sample data. It has better applicability for the diversity of drug molecular characteristics and less sample data. In this study, the SVR algorithm was used to quantitatively predict the inhibitory activity of Gal3 inhibitors. All data were plotted in 28-dimensional space, and the error tolerance parameter was set to 0.39.

#### Elastic Net Model

Elastic net (EN) is a linear regression model trained using L1, L2 norms as prior regular terms (Zou and Hastie, 2005). EN is very effective in cases where multiple features are interconnected. The cost function of elastic network is as shown in Equation 3. When r is equal to 0, it is RR, when r is equal to 1, it is Lasso regression (LR). EN is compromised in RR and LR.

(3) $$\begin{array}{r} J\left(\theta \right)\;=\;MSE\left(\theta \right)+\gamma \alpha \sum_{i=1}^{n}|\theta_{i}|+\frac{1-\gamma }{2}\alpha \sum_{i=1}^{n}\theta_{i}^{2} \end{array}$$

#### Random Forest Model

Random forest (RF) algorithm uses bootstrap method to generate training set (Breiman, 2001). Through random row and column (samples and features) sampling, seven decision trees were constructed to form a decision tree forest. The final prediction result was obtained by weighted average or voting. RF can achieve parallel learning and has a good filtering effect on noise and abnormal data (Athey et al., 2019). The model training process is given in Algorithm 2.

**Algorithm 2:** Random forest

1. Training set *D*_*f*_*feature*__ = {(*x*_1_, *y*_1_), (*x*_2_, *y*_2_), …, (*x*_*n*_, *y*_*n*_)}
2. For *t* = 1, 2, …, *T* do (1) Random sampling m sample points, constructing a training set *D*_*t*_ (2) Using *D*_*t*_ to train a decision tree (3) Least squares regression tree as an example Choosing the optimal segmentation variable i and the Segmentation Point s: $\begin{array}{l} \min_{i,s}[\min_{c_{1}}\sum_{x_{j}\in \;R_{1}(i,s)}{\left(y_{j}-c_{1}\right)}^{2}\\ \;+\min_{c_{2}}\sum_{x_{j}\in \;R_{1}(i,s)}{\left(y_{j}-c_{1}\right)}^{2}] \end{array}$ The selected pairs (*i, s*) were used to divide the region and determine the corresponding output values: *R*_1_(*i, s*) = $\left\{x|x^{i}\leq s\right\},R_{2}\left(i,s\right)=\left\{x|x^{i}>s\right\}$ *c*_κ_ = $\frac{1}{N_{\kappa }}\underset{x_{j}\in R_{\kappa }\left(i,s\right)}{\sum }y_{j},x\in R_{\kappa },\kappa =1,2$ Continue to iterate steps a and b for satisfying the condition The input space was divided into *K* regions: *f*(*x*) = $\overset{K}{\underset{\kappa =1}{\sum }}c_{\kappa }I\left(x\in R_{\kappa }\right)$ end

#### Gradient Boosting Regression Model

Gradient boosting regression (GBR) is an algorithm that improves by learning from mistakes. Each calculation of it is to reduce the residual error of the previous time and establish a new model in the direction of the negative gradient (Chen et al., 2018b). The least square error was used as the loss function of regression prediction, and the number of decisions was set to 7. The sub-models are integrated as the final predictive model. The algorithm flow is given in Algorithm 3.

**Algorithm 3:** Gradient boosting regression

1. Initialize *F*_0_(*x*) = $\arg\;{\text{min}}_{\gamma }\;\sum_{i=1}^{n}L\left(y_{i},\gamma \right)$
2. For *m* = 1, 2, ⋯ , *M* : (1) For *i* = 1, 2, ⋯ , *N* compute pseudo-residuals: *r*_*im*_ = $-{\left[\frac{\partial L\left(y_{i},\;F\left(x_{i}\right)\right)}{\partial F\left(x_{i}\right)}\right]}_{F_{\left(x\right)}=F_{m-1\left(x\right)}}$ (2) Fit a base regression tree to the targets *r*_*im*_ giving terminal regions *R*_*jm*_, *j* = 1, 2, ⋯ , *J*_*m*_ (3) compute multiplier γ_*jm*_ : $= arg\;{\text{min}}_{\gamma }\;\underset{x_{i}\in R_{jm}}{\sum }L\left(y_{i},\;F_{m-1}\left(x_{i}\right)\;+\;\gamma \right)$ (4) Update: $F_{m}\left(x\right)=F_{m-1}\left(x\right)+\overset{J_{m}}{\underset{j=1}{\sum }}\gamma_{jm}I\;\left(X\epsilon R_{jm}\right)$
3. Output $\hat{f}\left(x\right)=f_{M}\left(x\right)$ end

#### K-Nearest Neighbor Model

K-nearest neighbor (KNN) randomly divides the matrix into a training subset and a test subset, and returns the divided samples and labels. The Euclidean distance between the samples was calculated and sorted according to the distance (Abdel-Basset et al., 2020). The average value of the k (*k* = 4) samples closest to the target to be predicted was selected as the regression prediction value of the new sample.

#### XGBoost Model

XGBoost (XGB) algorithm improves the accuracy of the algorithm by adding the number of decision trees (Lai et al., 2020). The detailed process is shown as Algorithm 4. We used the xgb.DMatrix function. The representation method of the data in libsvm is a sparse matrix, which is very suitable for a large number of features and sparse. When there are missing values in the sample, XGB can automatically learn the split direction.

**Algorithm 4:** XGBoost

1. Training set *D* = {(*x*_1_, *y*_1_), (*x*_2_, *y*_2_), …, (*x*_*m*_, *y*_*m*_)}
2. Loss function MSE (mean square error): $L\left(\theta \right)={\underset{i}{\sum }\left(y_{i}-{\overset{¯}{y}}_{i}\right)}^{2}$ where, *y*_*i*_ is the real value, ȳ_*i*_ is the predicted value.
3. For *k* = 1, 2, …, *K* do (1) The definition of the predicted model: ${ȳ}_{i}=\overset{K}{\underset{k=1}{\sum }}f_{k}\left(x_{i}\right),f_{k}\in F$ K is the number of trees, F includes all possible trees, *f*_*k*_ is a specific tree, *f*_*k*_(*x*_*i*_) represents the predicted value of *x*_*i*_ on the kth tree. (2) Minimizing objective function *L*_*obj*_(θ) to acquire *f*_*k*_. $L_{obj}\left(\theta \right)=\overset{n}{\underset{i}{\sum }}L\left(\theta \right)+\overset{K}{\underset{k=1}{\sum }}\Omega \left(f_{k}\right)$ Ω(*f*_*k*_) is the complexity of tree *f*_*k*_.
4. Output *f*_*k*_ e n-dimensional d

#### Deep Belief Network

**Figure 4:**
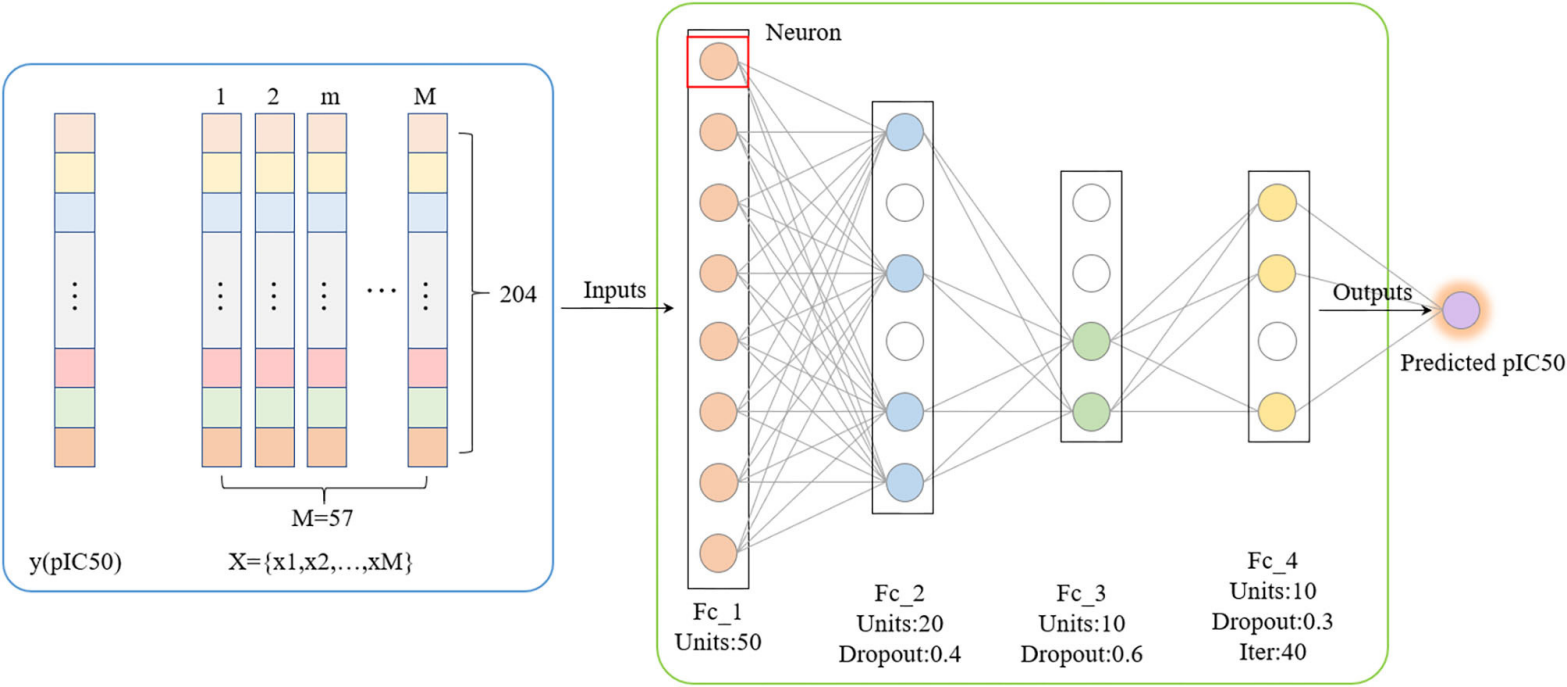
Deep Belief Network generated by applying dropout. The mean square error is used as the loss function. The neural network weights are updated by an Adam optimizer with a learning rate of 0.0006. Rectified Linear Units and Dropout methods are utilized.

Compared with traditional artificial neural networks, deep learning-based frameworks with huge numbers of multiple hidden layers maintain two-way fidelity of information transferring between different levels of abstraction during model learning (LeCun et al., 2015). Deep learning algorithms are widely used in drug design research such as protein structure prediction and disease diagnosis (Li et al., 2020; Senior et al., 2020). Deep belief networks (DBN) is the foundation of deep learning. In this study, we trained a quantitative prediction model of the inhibitory activity value of Gal3 inhibitors using a simple fully connected neural network with three hidden layers (Figure 4). The activation function was configured as rectified linear unit (ReLu) (Agarap, 2018). Furthermore, we introduce the Dropout method was to reduce the amount of calculation and increase the robustness of the model, by randomly pruning some neural units in the hidden layer with a predetermined probability (Zhang et al., 2018).

### Molecular Dynamics Simulation

Molecular docking methods can narrow the scope of searches from large compound databases. Combining the molecular docking score and the target value predicted by artificial intelligence-based models, four optimal compounds (6318, 5372, 22157, 7649) ligands were finally screened as candidates for MD simulation. MD simulation can simulate and analyze the ligand-receptor movement process, and obtain some key information from the process to verify their stability (Song et al., 2019). SwissParam was chosen as the tool for generating topology files (Zoete et al., 2011), the mol2 file of candidates were submitted to obtain topology files with parameters such as atom type, charge and bonding conditions. The four candidate small molecules were combined with the processed Gal3 receptor protein and divided into four groups A, B, C, and D for MD simulation. CHARMM27 force field was used to describe the receptor protein (Sapay and Tieleman, 2011). Using TIP3P water molecule model, adding water as the solvent of the complex system, while adding NaCl and sodium ions to maintain the electrical neutrality of the system. The steepest descent method was used to optimize the energy of 5,000 steps to make the system reach near the lowest point of energy. After the optimization, it entered the equilibrium stage, and the positions of proteins and ligands needed to be restricted. First, balance for 10 ns under the constant number of atoms, constant volume and constant temperature (NVT) ensemble, and the system temperature rose from 0 to 310 K. Then it was equilibrated for 10 ns under the constant number of atoms, constant pressure and constant temperature (NPT) ensemble, and the temperature was kept at 310 K, which was used to simulate the physiological environment in the human body. The V-rescale method is used for temperature coupling, and the Parrinello-Rahman method is used for pressure coupling. After the equilibrium stage was over, the restriction was released, and each combination was subjected to 100 ns MD simulation under the NPT ensemble. In the simulation process, periodic boundary conditions (PBC) were used in all directions to eliminate possible boundary effects. The time step was set to 2fs, and the coordinate file and the energy file were recorded every 2ps.

## Results and Discussion

### Molecular Docking

**Figure 5:**
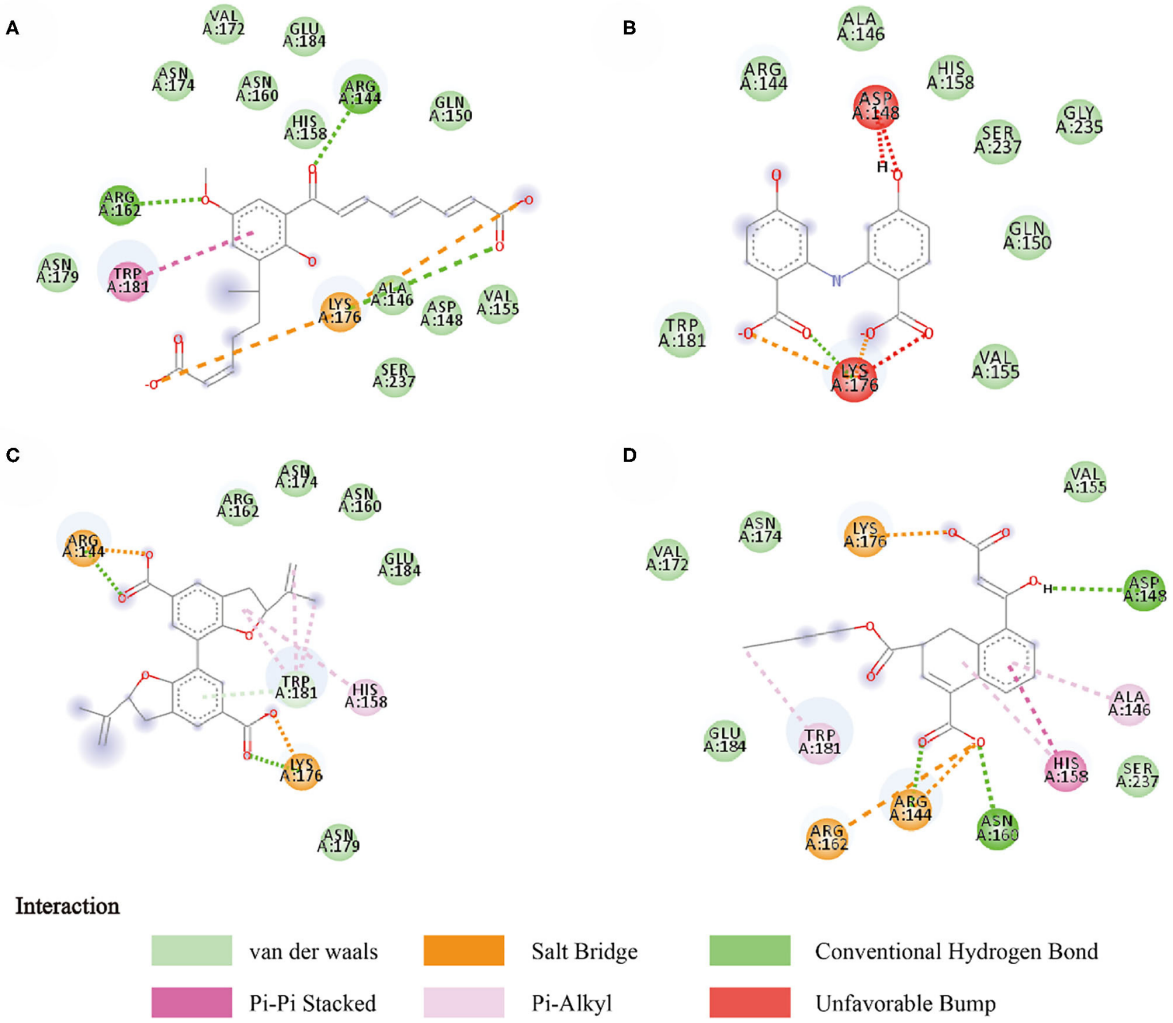
Two-dimensional image of molecular docking results. **(A)** 6318, **(B)** 2007_5372, **(C)** 2007_7649, **(D)** 2007_22157.

**Figure 6:**
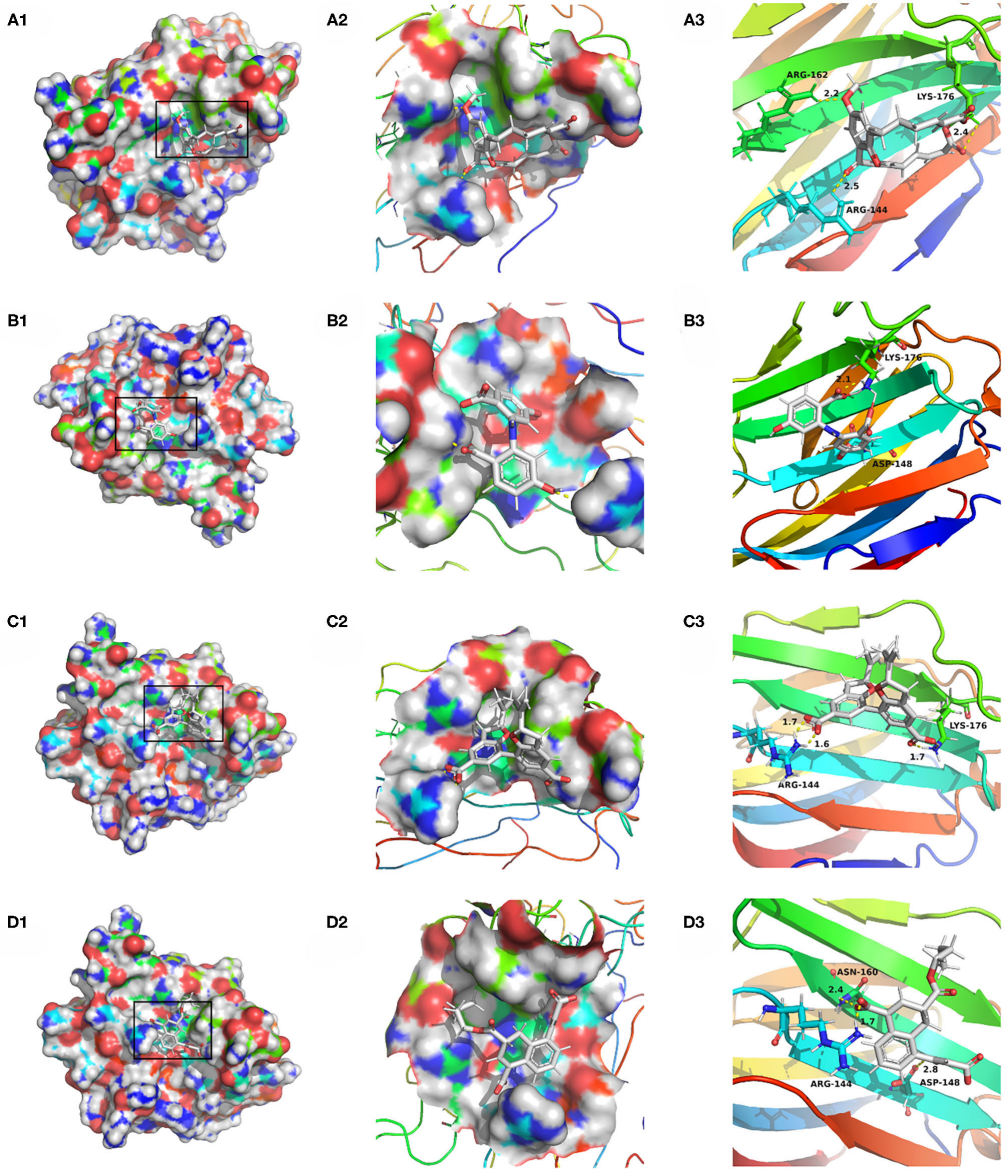
Docking pose of **(A)** 6318, **(B)** 2007_5372, **(C)** 2007_7649, **(D)** 2007_22157.

Compounds from the TCM database were used for high-throughput virtual screening against Gal3, and the top 10 compounds were shown in Table 1. According to the docking scores of screenings, 6318, 5372, 7649, and 22157 (Table 1) were selected for further analysis. 2D diagram of these molecules in docking results showed potential interactions between key residues and ligands, including hydrogen bonds, van der Waals, salt bridge, Pi-Pi stacked, etc (Figure 5). These four TCM compounds have a common property, a carboxylate group in their chemical structures. Based on the docking poses of Gal3 in Figure 6, the α, β-unsaturated carbonyl groups of 6318 formed two hydrogen bonds (2.4 and 2.5 Å) with LYS176 and ARG144 of Gal3, respectively. The methoxy of 6318 formed a hydrogen bond (2.2 Å) with ARG162. The carboxylate group of 5372 formed a 2.1 Å hydrogen bond with LYS176. 7649's benzoic acid groups interacted with LYS176 and ARG144 through three hydrogen bonds (1.7, 1.6, and 1.7 Å). The carboxylate group of 22157 had H-bonding interactions with ARG144 and ASN160 (1.7 and 2.4 Å), and the β-ketone carboxyl group engaged with ASP148 and LYS176 through two H-bonds. The hydrogen bond interaction of the compounds with LYS176 and ARG144 indicated that these were two key residues. The same experiment had been done with ZINC database and the results showed that the binding affinity was weaker than that from the TCM database.

**Table 1:** Docking score, predicted activity value of top ten TCM candidates, top two ZINC candidates and control ligand.

**Index**	**Name**	**Predicted value (pIC50)**									**Docking Score**
		**ABR**	**RR**	**SVR**	**EN**	**RF**	**GBR**	**KNN**	**XGB**	**DBN**	
6318	Chinese Hawthorn	7.119	6.830	6.791	6.851	6.665	6.839	6.693	7.093	6.162	125.276
22157	Longbract Cattail Pollen	7.155	7.519	7.532	7.440	6.696	6.937	6.554	7.093	5.743	116.132
5372	Carnation	6.951	7.023	7.148	7.128	7.061	7.037	6.887	6.380	5.591	119.682
7649	Staphyleaceae	6.650	7.956	8.281	8.099	6.886	6.779	6.942	6.510	6.629	116.693
2246	Amur Adonis	6.951	6.873	6.967	7.012	6.816	7.002	6.938	6.380	5.537	117.959
14992	White Mulberry Fruit	5.658	4.901	4.750	4.933	5.768	6.088	5.838	5.929	2.528	114.298
2670	Java Brucea	5.793	4.861	4.774	4.926	5.849	6.444	6.257	5.598	3.829	106.878
8713	Whiteflower Leadword	7.143	7.048	7.057	7.083	6.726	6.937	6.552	7.211	2.044	110.309
22676	Common Threewingnut	6.076	8.094	7.947	7.853	5.767	5.723	6.177	5.866	3.541	110.031
210	Fresh Common Ginger	6.509	5.143	5.171	5.100	6.347	6.011	6.020	6.769	2.963	106.732
ZINC000019363537	Tetraethylenepentamine	5.301	4.622	4.630	4.538	5.319	5.133	5.742	5.438	0.611	102.451
ZINC000019364225	Trientine	5.818	5.455	5.276	5.313	5.577	5.836	5.759	5.598	0.529	90.915
Control	J4N	6.180	6.838	6.902	6.899	6.542	6.034	6.979	6.510	4.962	56.381

### Prediction Results of Models

During the whole validation process, the prediction accuracy on testing sets is used to quantify the performance of different prediction algorithms. Both Mean Square Error (MSE) and square correlation coefficient (R-square) are set as the metric for prediction accuracy. MSE is the expectation of the square of the difference between the predicted value and the true value, which is used to evaluate the predicted result. The smaller the MSE, the stronger the model's ability to fit the experimental data. R-square represents the quality of a fit through changes in data. The normal value range is 0 to 1. The closer to 1 indicates that the variable of the equation (input feature) has a stronger ability to explain Y (pIC50). Table 2 lists the R square and MSE values of all prediction models.

**Table 2:** R-square and mean squared error (MSE) values of all trained models.

	**ABR**	**RR**	**SVR**	**EN**	**RF**	**GBR**	**KNN**	**XGB**	**DBN**
R2_train	0.990	0.869	0.850	0.851	0.920	0.991	1.000	0.970	0.935
R2_test	0.923	0.795	0.848	0.837	0.848	0.859	0.770	0.901	0.900
MSE_train	0.002	0.056	0.064	0.064	0.036	0.001	0.000	0.014	0.025
MSE_test	0.027	0.087	0.064	0.069	0.052	0.049	0.080	0.034	0.045

#### Feature Selection

**Figure 7:**
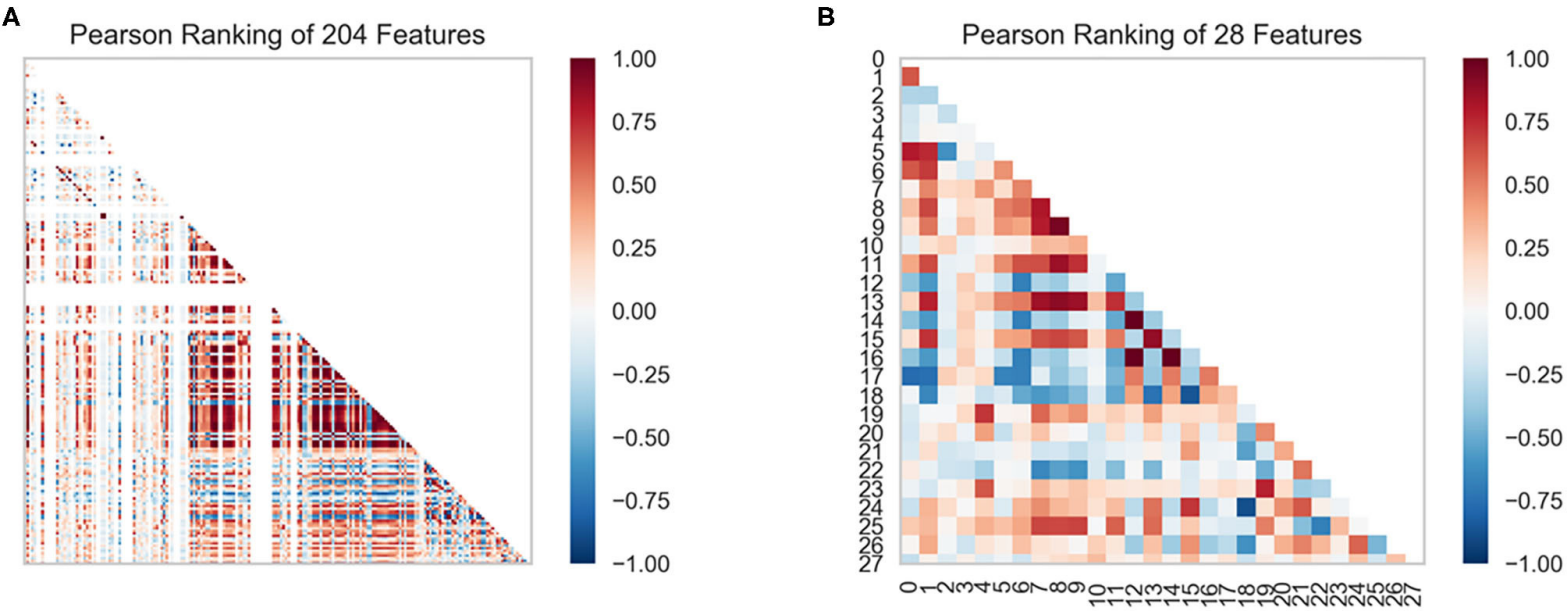
Feature ranking results are derived from the Pearson algorithm. **(A)** 204 features, **(B)** 28 features.

**Figure 8:**
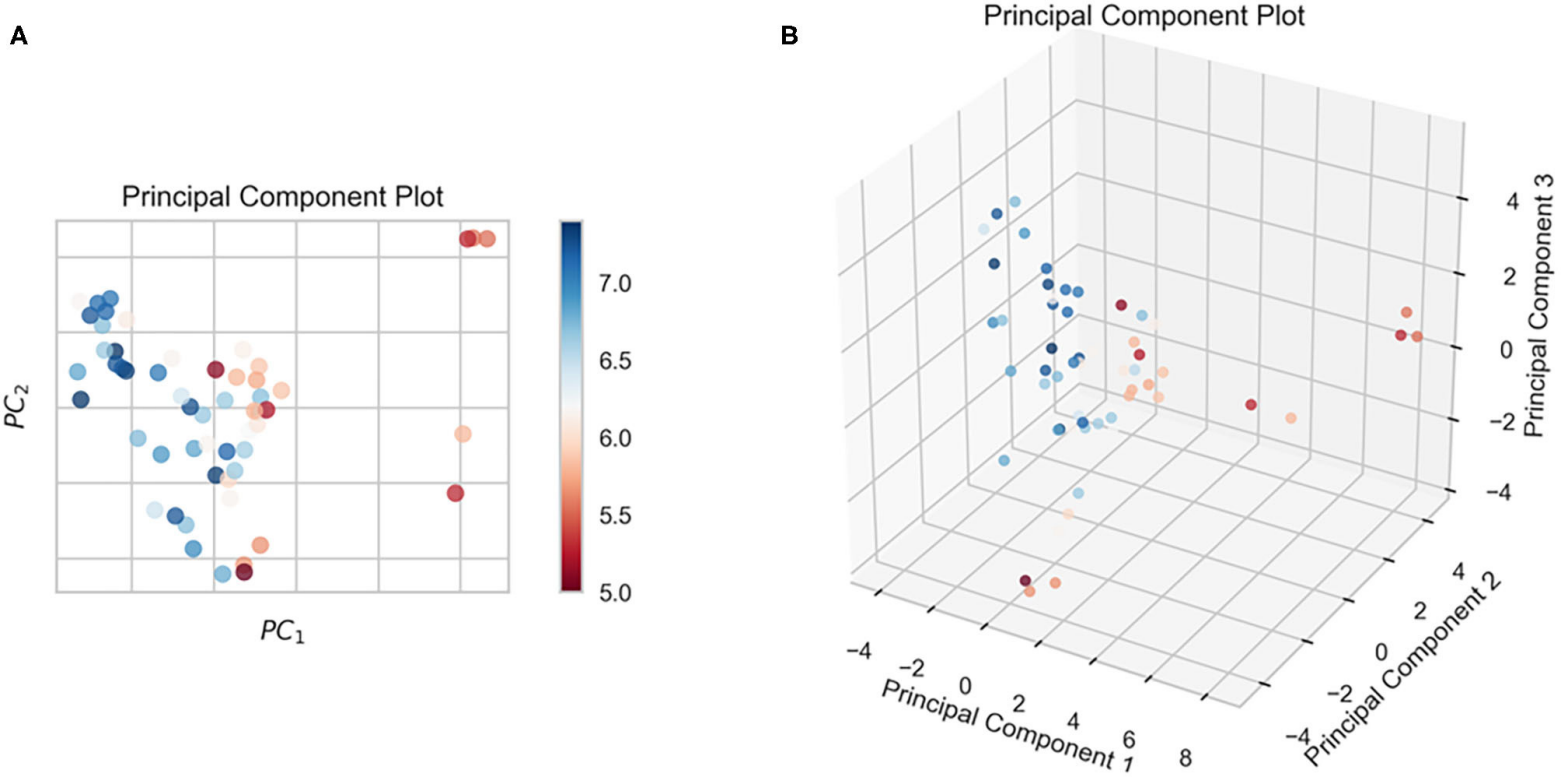
The results of the n-dimensional principal component analysis. **(A)** 2D PCA, **(B)** 3D PCA.

**Figure 9:**
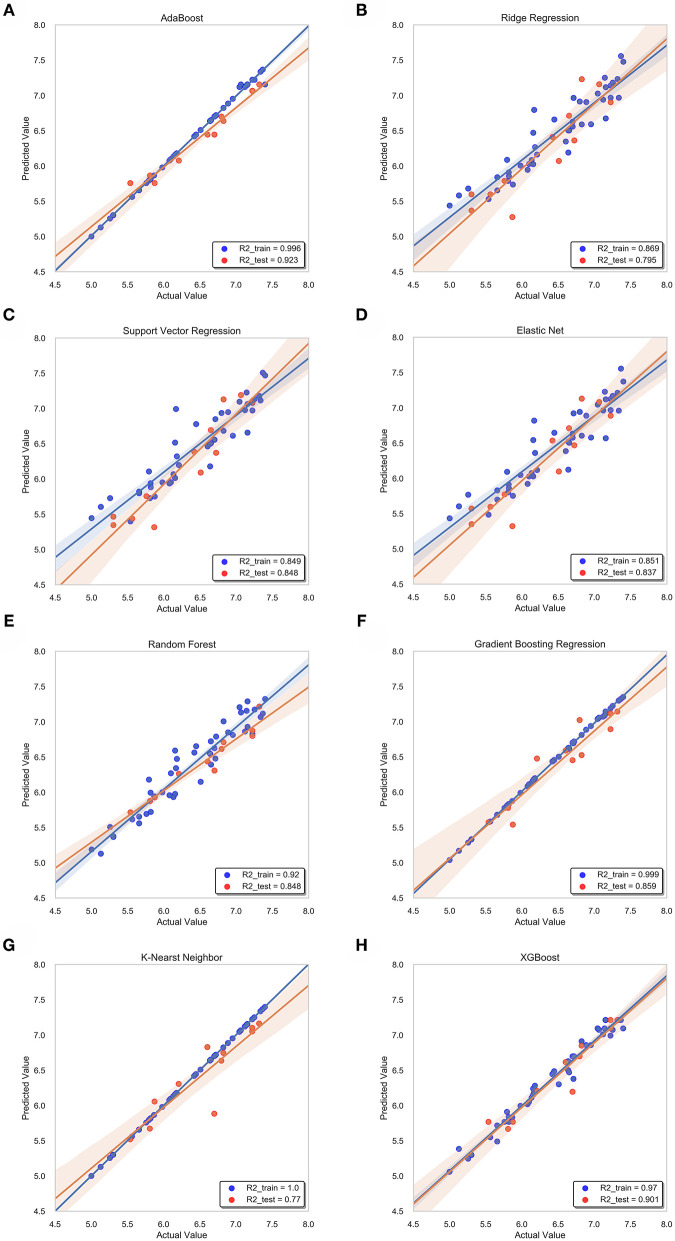
Correlation between predicted value and actual value of machine learning model. Training set (blue dots) and testing set (red dots) are shown. The confidence interval is 95%. **(A)** ABR, **(B)** RR, **(C)** SVR, **(D)** EN, **(E)** RF **(F)** GBR, **(G)** KNN, **(H)** XGB.

In this study, we obtained only 56 samples, but the feature dimension is as high as 204. Theoretically, this can easily lead machine learning models to get over-fitting. We used the following methods to perform feature dimensionality reduction to search for representative features. Firstly, Pearson correlation coefficient has very good applicability in the characterization of correlation. Figure 7A is a heat map of Pearson correlation coefficients between all features, the deeper the red, the stronger the correlation. It can usually be considered to have a strong correlation when the correlation is >0.9, and parameters would be eliminated. Then, principal component analysis (2D PCA and 3D PCA) achieved dimensionality reduction by integrating the original single variable to obtain a new set of comprehensive variables (Figures 8A,B). What's more, the elements with sample feature variance <0.05 were eliminated by calling the Variance Threshold library function, and the Lasso function was used to select the variables of the sample data based on the penalty method. Finally, the original coefficients were compressed, and the insignificant variables were directly discarded. Figure 7B is a heat map of Pearson correlation coefficients between the remaining 28 features after eigenvalue preprocessing. These 28 indicators were set as input features for all involved machine learning models. Through a 5-fold cross-validation method, the predictive ability of the sample model is evaluated. Correlation between predicted values and actual values (pIC50) of ABR, RR, SVR, EN, RF, GBR, KNN, XGB models were shown in Figure 9.

#### RR and EN Model

For ridge regression, elastic net and lasso regression models, model regularization is introduced to reduce the over-fitting, but the method of restraining weight is different. The R-square of the RR model and the EN model on the training sets were 0.87 and 0.85, respectively, but the R-square of the EN model on the test sets was 0.84, which was significantly better than 0.79 of the RR model (Figures 9B,D). This might be because only a few key features are related to pIC50 in all features, and the RR model retains those irrelevant features, which increases the fitting error. In this study, the number of features was much larger than the number of samples. The EN model could reduce the weight of non-key features to zero, and only retained a few key features, so it showed better fitting results.

#### ABR, RF, GBR, and XGB Model

The R-square of the ABR, RF, GBR, and XGB models on the training sets were all higher than 0.9 (Table 2), which was better than the RR and EN models. This was because they all used integrated learning methods and used different strategies to combine individual learners into a committee, which improved the generalization ability of the model. Based on bagging integration method, the prediction result of RF was obtained by parallel calculation of all decision trees. It made the model susceptible to large deviations due to the influence of individual wrong decision trees, so the R-square on the training sets was only 0.92 (Figure 9E). The R-square of the ABR and GBR models on the training sets were both close to 1 (Figures 9A,F). This was most likely because they used the Boosting method to upgrade the weak learner to a strong learner and the sample size was relatively small. ABR continuously optimized the sample weights of each round of training, the trained model with the testing sets mean square error (MSE) of 0.027 and R-square of 0.92 (Table 2), which showed the best performance. XGB added a regular term to the cost function that reduced the variance of the model and avoided overfitting. With the help of the variable importance indicator feature_importances_, we obtained the weight of the importance of the XGB model variable. It could be seen that ALogP_MR and ES_Count_aaaC were the top two eigenvalues. The distribution of predicted values around actual values of the XGB model were plotted in Figure 9H, and the R-square on the training sets and the test sets reached 0.97 and 0.9, respectively.

#### SVR and KNN Model

Before ensemble learning and neural network algorithms showed superior performance, SVM algorithm basically occupied a dominant position, especially in the field of classification. For Gal3 inhibitor studies, there were actually fewer samples available for reference. Even so, since SVR basically did not involve probability measurement and the law of large numbers, the model still showed high prediction accuracy, and the R-square on the test sets was close to 0.85 (Figure 9C). However, the shortcoming of SVR was also exposed. When the feature dimension was much larger than the number of samples, the SVR model tended to ignore the correlation of mutual characteristics, so the R-square on the training sets was only 0.85. After repeated calculation and verification, the linear kernel was finally selected, and the error tolerance was set to 0.39 to ensure that the model had sufficient generalization ability to avoid overfitting. The fitting curve of the KNN model was perfect, and the R-square on the training sets had reached 1, which was suspected of overfitting. The number k of favorable features was filtered through the built-in function of Scikit-learn, and it was verified that the model performs best when k was equal to 4. When the independent variable dimension was small, the KNN model could significantly reduce the error. But as the variable gradually increased, the mean value of the dependent variable corresponding to the closest value to the target might deviate from the actual value exponentially. The above reasons caused the model accuracy to be significantly reduced. The R-square on the test sets was only 0.77, which was the worst performance among all models (Table 2).

#### Deep Belief Network

**Figure 10:**
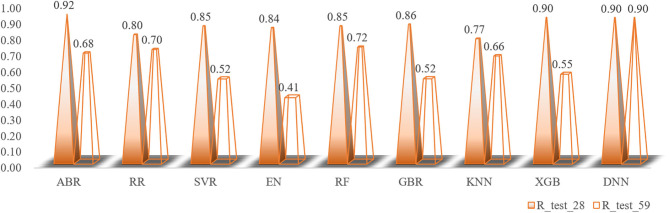
The changes in the R-square values of all models when the number of feature variables is increased.

The total number of sample data we obtained was only 56, far less than the number of 204 feature dimensions. In view of this situation, traditional machine learning models usually removed most of the features during the preprocessing process. Although a model with higher prediction accuracy could be obtained, this actually consumed the credibility of the model. DBN could avoid this problem as much as possible, allowing more features to participate in training. Although it took thousands of times the training time, the trained model had better prediction reliability and more convincing. Using the Dropout method, the dropout rates of the first, second and third layers of the neural network were 0.4, 0.6, and 0.3, respectively, and a total of 500 modeling attempts were made. The R-square of the best trained model on the training sets and test sets were 0.94 and 0.9, respectively. We had tried in the previous machine learning model. When the parameter threshold was set to 0.01 and alpha was set to 0.001, 59 features are retained. However, the accuracy of all models was significantly reduced. The R-square on some model test sets was even lower than 0.5 (Figure 10). Through comparison, it could be found that the DBN model still showed high accuracy under low sample set and high feature dimension.

### Molecular Dynamics Simulation

**Figure 11:**
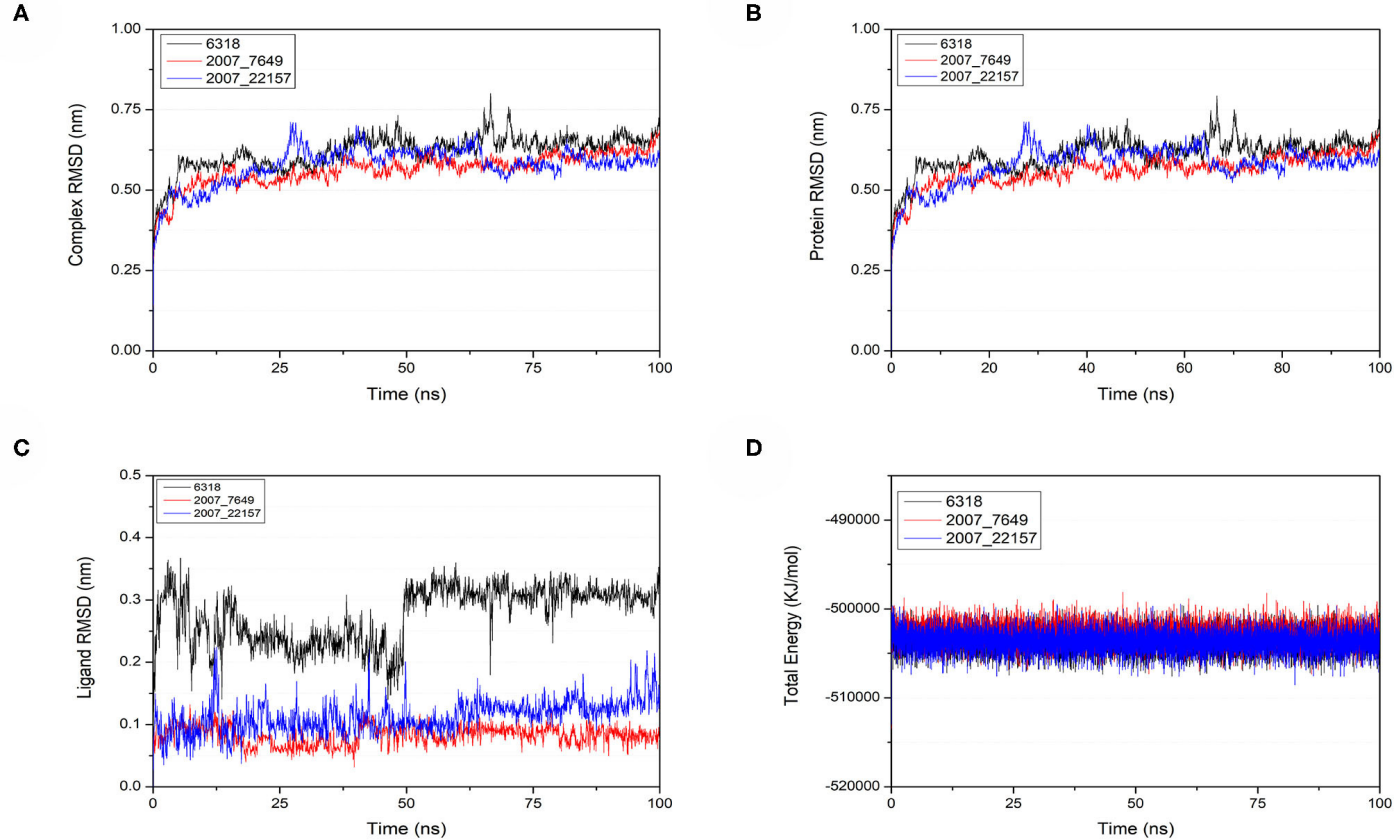
Changes of RMSD and total energy in molecular dynamics simulation. **(A)** RMSD changes of complex, **(B)** RMSD changes of protein, **(C)** RMSD changes of ligands, **(D)** Total energy changes between protein and ligands.

**Figure 12:**
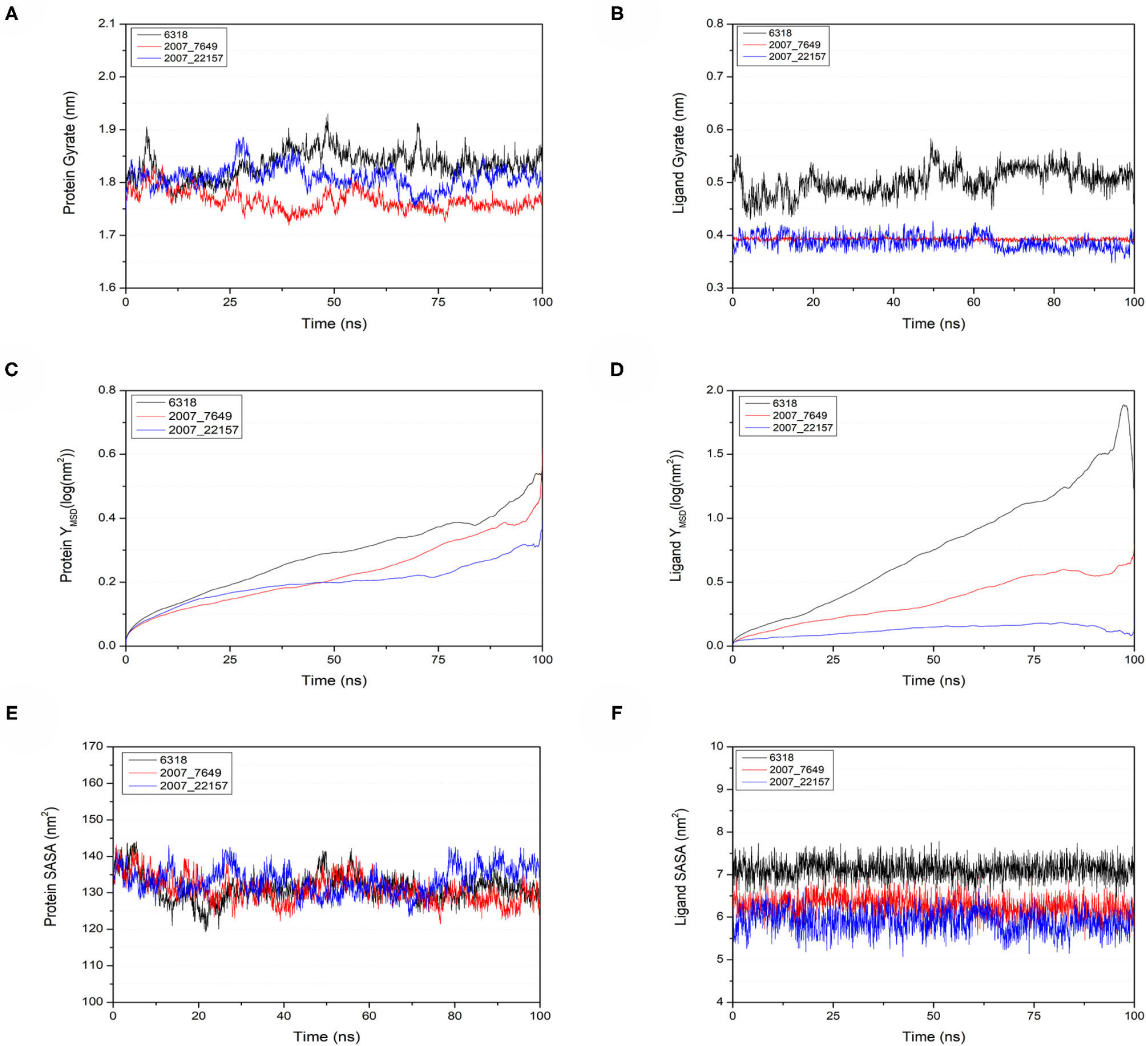
Gyrate, MSD, and SASA changes of proteins and ligands in molecular dynamics simulations. **(A)** Gyrate of protein, **(B)** gyrate of ligands, **(C)** MSD of protein, **(D)** MSD of ligands, **(E)** SASA of protein, **(F)** SASA of ligands.

**Figure 13:**
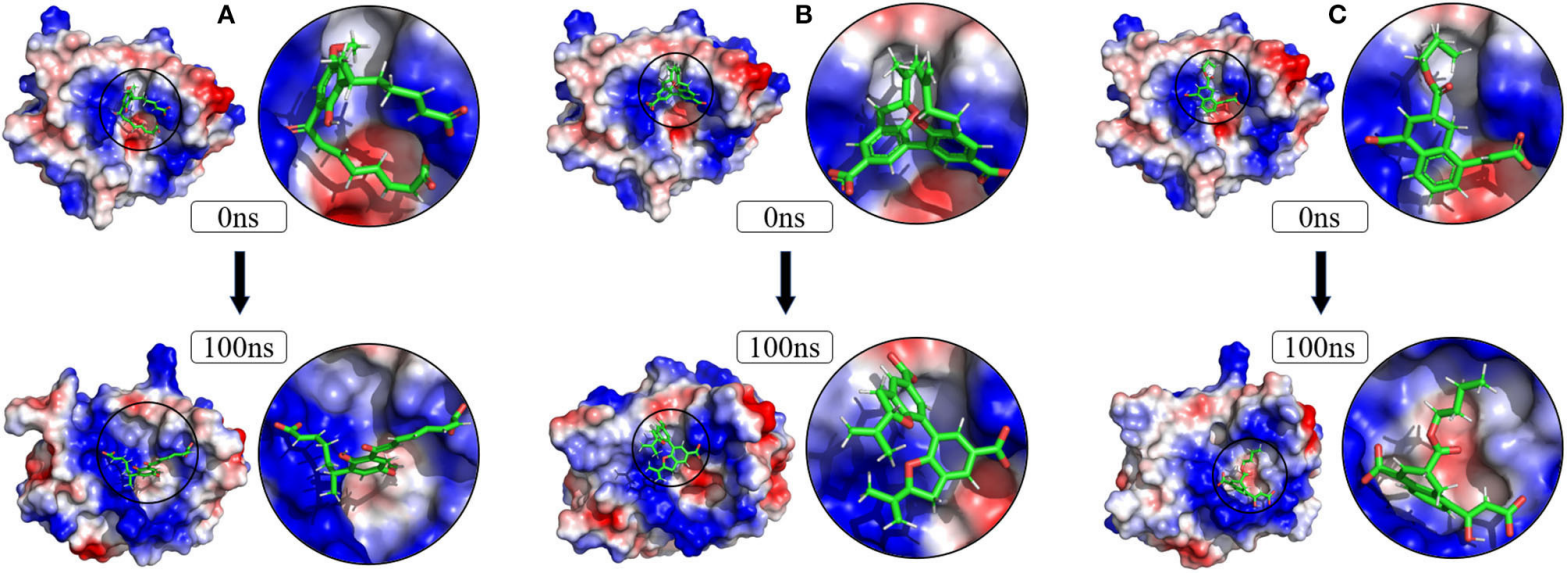
Changes of the binding posture during MD in microenvironment. Although the conformation of the ligands changed, the position where the ligands bind to the protein pocket does not change. **(A)** 6318, **(B)** 2007_7649, **(C)** 2007_22157.

To verify the binding stability of the receptor-ligand complexes, we performed molecular dynamics simulations in 100 ns with Gromacs 2018 software (Kutzner et al., 2019). Unfortunately, 5372 was detached from the binding site during the molecular dynamic simulation, although it showed good results in the molecular docking and AI-based models prediction process. The escape of 5372 indicated that its binding stability to Gal3 was poor. The RMSD was calculated to evaluate the deviation of the structure from the original starting structure over the course of the simulations. In the RMSD results (Figures 11A–C), the RMSD values of the three candidates and Gal3 complexes shown an upward trend at initial 10 ns, then tended to stabilize with a relatively flat curve and maintained around 0.5–0.6 nm. The protein RMSD change curve is similar to the complex RMSD change curve. Based on ligand RMSD, 6318 had higher fluctuation rate than other candidates, which may explain high ligand gyration value. The RMSD results suggested that candidates 7649 and 22157 have higher binding stability to Gal3 protein. Besides, the total energy of simulation systems in the 100 ns process was calculated to analyze the energy changes in the complexes. And the results shown that the energy of protein-ligand complexes was stable and it had been maintained between about −510 000 to −500 000 kJ/mol (Figure 11D). The radius of gyration could give a measure of the compactness of the structures, and can also give a measure of the atomic mass relative the molecular center mass. As shown in Figures 12A,B, protein gyrate and ligands gyrate were stable in general during the MD simulation process, and the gyrate of 6318 was higher than other two ligands (include target protein system), which was consistent with the RMSD results. MSD revealed the movement of atoms from the initial position to the final stage of MD simulation, indicating the movement trend of each ligand or protein. The low and stable MSD value of the ligand shows the stability of the binding, while the decrease of the MSD value indicates that the ligand may be close to the binding pocket. The extremely high MSD value and the increasing MSD value mean the ligand has a tendency to escape. In the process of the whole simulation (Figures 12C,D), although the MSD values of Gal3 and three ligands both increased, the changes of all measured MSD values maintained in a low range. It was worth noting that 6318 obtained much higher ligand MSD value compared to other two ligands, which indicates the ligand 6318 have a trend of escaping from the binding pocket. From the SASA calculated results, we can analyze the hydrophilicity and hydrophobicity of the simulation system. The solvent accessible area of Gal3 decreased significantly from 0 to 15 ns, and then remained to a relatively stable area (Figure 12E). Meanwhile, as shown in Figure 12F, the SASA values of all ligands were very stable in the simulation process. This can further indicate the stability of the complex systems. Finally, the binding postures of three ligands and target protein in the initial and final conformations were displayed in Figure 13. The ligands rotated in the same pocket but not detached from the target protein.

## Conclusion

The new discovery of Gal3 in the pathogenesis of HD provides a new target and new method for ND treatment. In order to find potential inhibitors of Gal3, we have completed the following work and obtained satisfactory results. Using molecular docking methods, we initially screened a batch of small molecules with relatively stable docking from a large drug molecule library. Multiple artificial intelligence-based models were constructed, and known Gal3 inhibitors were used as sample sets to train the models. From the performance parameters of the model, all models achieved high overall accuracy sensitivity. The R-square of XGBoost model on the test sets was higher than other algorithms, and there was no overfitting on the training sets. We not only screened from the TCM database, but also used the ZINC database to do the same. The results showed that the molecules from the TCM database performed better than the ZINC database in terms of binding stability and pIC50 value predicted by AI models. Comparing the prediction results of all models, we completed further screening and narrowed the candidate range. Finally, through MD simulation, we further verified the stability of the complexes, the final candidate ligand and the target protein complexes showed stable binding throughout the simulation time. Combining all experimental results, the active ingredients 1,2-Dimethylbenzene and Typhic acid contained in *Crataegus pinnatifida* and *Typha angustata* may become the new drug formulation for ND treatment. We provide a new strategy with applying AI-based methods to the drug screening process, which can greatly reduce the cost of new drug development. Screening drug molecules from the TCM database is an innovation and beneficial supplement to screening from the general database. In summary, this study has explored a highly accurate integrated architecture to reduce the drug screening process. With the application of artificial intelligence, medical practitioners exclude candidates with low probability based on prediction results, which reduces the risk of downstream decision-making for better resource planning and allocation. The proposed integration method shows high accuracy under different algorithm models, indicating that artificial intelligence-based drug development has application prospects. Artificial intelligence-based application is an improvement and supplement to the existing traditional drug screening based on molecular interaction relationships.

## Data Availability Statement

The raw data supporting the conclusions of this article will be made available by the authors, without undue reservation.

## Author Contributions

SJ and CC designed research. LD, WZ, LZ, XH, and ZL worked together to complete the experiment and analyze the data. SJ, CC, LD, and WZ wrote the manuscript together. All authors contributed to the article and approved the submitted version.

## Conflict of Interest

The authors declare that the research was conducted in the absence of any commercial or financial relationships that could be construed as a potential conflict of interest.
